# Supplementary material for: Universality, criticality and complexity of information propagation in social media
Source: Nat Commun. 2022 Mar 14;13:1308. doi: 10.1038/s41467-022-28964-8 (PMC8921196; doi:10.1038/s41467-022-28964-8)
Supplement: Supplementary file 1 — Supplementary Information [file 41467_2022_28964_MOESM1_ESM.pdf]

Supplementary Information: Universality, criticality and complexity of information propagation on social media

Daniele Notarmuzi,[1] Claudio Castellano,[2] Alessandro Flammini,[1]
Dario Mazzilli,[1,3] Filippo Radicchi[1*]

[1]Center for Complex Networks and Systems Research,
Luddy School of Informatics, Computing, and Engineering
Indiana University, Bloomington, Indiana 47408, USA
[2]Istituto dei Sistemi Complessi (ISC-CNR),
Via dei Taurini 19, I-00185 Roma, Italy
[3]Centro Fermi
Via Panisperna 89 A, Roma, Italy.
*To whom correspondence should be addressed; E-mail: filiradi@indiana.edu

A Data cleaning

Table S1 summarizes the properties of the empirical data we analyzed in the paper.

Fig. S1 shows the daily rate of activity in each data set. While TWT and WEI display a rate of activity almost constant over our observation period, the other data sets display significant variations.

We restrict our attention to observation windows where all data are nearly stationary, i.e., the number of events per unit time is roughly constant for time units much larger than the temporal resolution of the data. These shorter observation windows are highlighted in Fig. S1.

Daily rates in the reduced temporal window are shown separately in Fig. S2. Table S2 reports information about the data sets as they result after reducing the temporal windows. The

Data set	Acronym	Temporal window	Time series	Events	URL
Twitter	TWT	Oct. 1, 2019 - Nov. 30, 2019	15,700,708	710,124,693	[1]
Telegram	TLG	Sep. 22, 2015 - Jun. 11, 2019	5,141,612	75,596,578	[2]
Parler	PARL	Aug. 1, 2018 - Jan. 11, 2021	183,062,974	22,831,777	[3]
Weibo	WEI	Jan. 2, 2012 - Dec. 30, 2012	1,958,768	19,560,710	[4]
StackOverflow	STCK	Aug. 1, 2008 - Dec. 1, 2019	56,525	55,084,783	[5]
Delicious	DEL	Mar. 10, 2007 - Aug. 10, 2011	1,052,098	21,373,192	[6]

Table S1: Summary table of the data. From left to right we report: the name of the data set, the acronym we use to refer to the data set, the temporal window of data collection, the total number of time series, the total number of events(times) and a link to the original data. Events correspond to the observation of items in the original data.

results shown in the main text and in the Supplementary Information (SI) are all obtained from the analysis of data sets over reduced observation windows. Table S3 reports the values of the optimal resolution Δ^* obtained by means of the percolation analysis.

Data set	Acronym	Temporal window	Time series	Events	URL
Twitter	TWT	Oct. 1, 2019 - Nov. 30, 2019	15,700,708	710,124,693	[1]
Telegram	TLG	1,350 days	4,972,879	72,593,735	[2]
Parler	PARL	204 days	753,215	20,634,978	[3]
Weibo	WEI	Jan. 2, 2012 - Dec. 30, 2012	1,958,775	20,365,986	[4]
StackOverflow	STCK	2,639 days	55,802	45,227,132	[5]
Delicious	DEL	700 days	528,170	7,892,075	[6]

Table S2: Summary table of the data after reduction of the observation windows. From left to right we report: the name of the data set, the acronym we use to refer to the data set, the temporal window of data collection, the total number of time series, the total number of events (times) and a link to the original data.

Data set	TWT	TLG	PARL	WEI	STCK	DEL
Δ^* (s)	1,566	30,549	3,845	8,413	21,135	29,853

Table S3: Summary table of the values of Δ^* obtained by maximizing the susceptibility on data sets generated on social media. We report the name of the data set (upper row) and the associated value of Δ^* (bottom row), expressed in seconds.

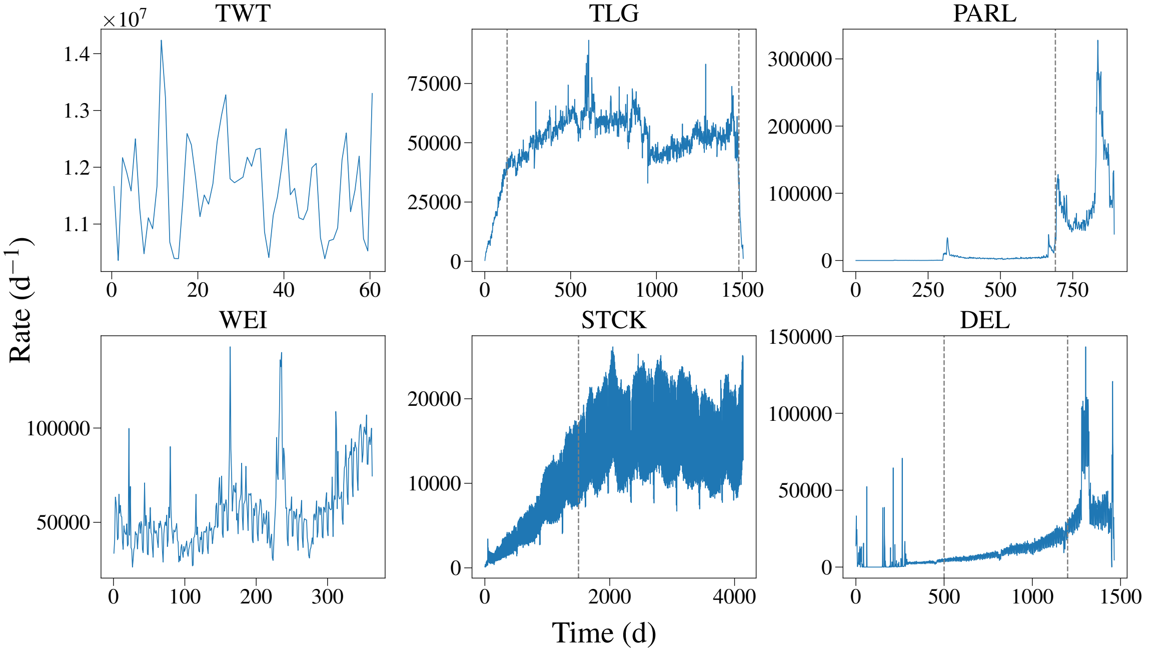

Figure S1: Daily rate of activity in the original data sets. The rate is computed as the total number of events per day. The dashed vertical lines in the panels for TLG and DEL mark the beginning and the end of the reduced temporal window. The dashed vertical line in the panels for PARL and STCK mark the beginning of the reduced temporal window, which in this case ends where the original window ends.

B Beyond social media: neuronal systems and earthquakes

In addition to the six data sets concerning social media, we further study data sets describing activity in different systems.

We consider a set of 88 time series, collected in Ref. [7], generated by monitoring the spontaneous activity of dissociated cultures of rat's hippocampal cells. Specifically, we consider the culture number 1 in the 11-th day in vitro and refer to it as RHDC (Rat Hippocampal Dissociated Cultures). A set of 166 time series, collected in Ref. [8, 9], generated by monitoring the neural activity in cultured slices of mice somatosensory cortex is further considered. In this case we consider the data set number 1 and refer to it as MSOS (Mouse Somatosensory

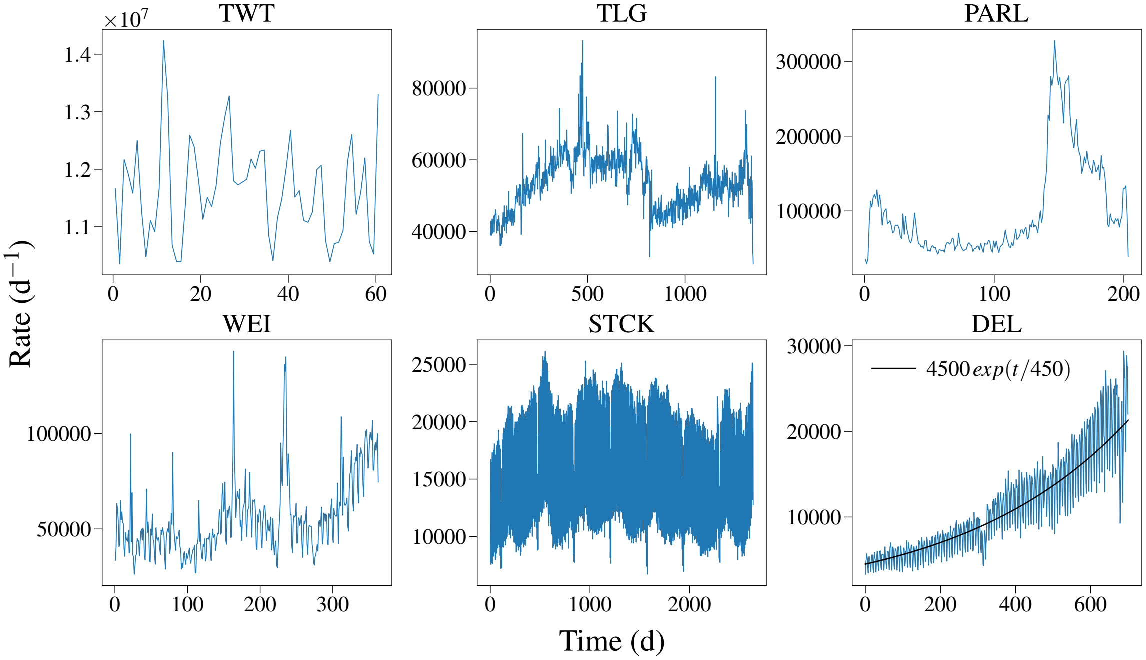

Figure S2: Daily rate of activity in the reduced data sets. The rate is computed as the total number of events per day. In each panel we show the same data as in Fig. S1, but restricted to the temporal windows delimited by the dashed vertical lines respectively for each data set.

Organotypic Slice). We also consider a data set generated by monitoring the neural activity in the premotor cortex of a macaque, collected in Ref. [10]. We use the MT_S2 data set and refer to it as MPC (Macaque Premotor Cortex). In these systems each electrode is associated to a time series and an event corresponds to the detection of a spike by the electrode.

We further consider three catalogues of earthquakes reporting seismological activity in Japan [11], in California [12] and in Europe [13]. In the case of the California catalogue, we discard all events prior to Jan. 1, 1900. For each of these catalogues, we divide geographical space into bins. For each bin, we construct a time series composed of the time of events whose longitude and latitude falls within the bin, in the same way as done in Ref. [14]. The procedure of geographical binning is illustrated in Fig. S3. Table S4 summarizes the properties of these data sets. Table S5 reports the values of the optimal resolution Δ^* for the data sets not

concerning activity in social media.

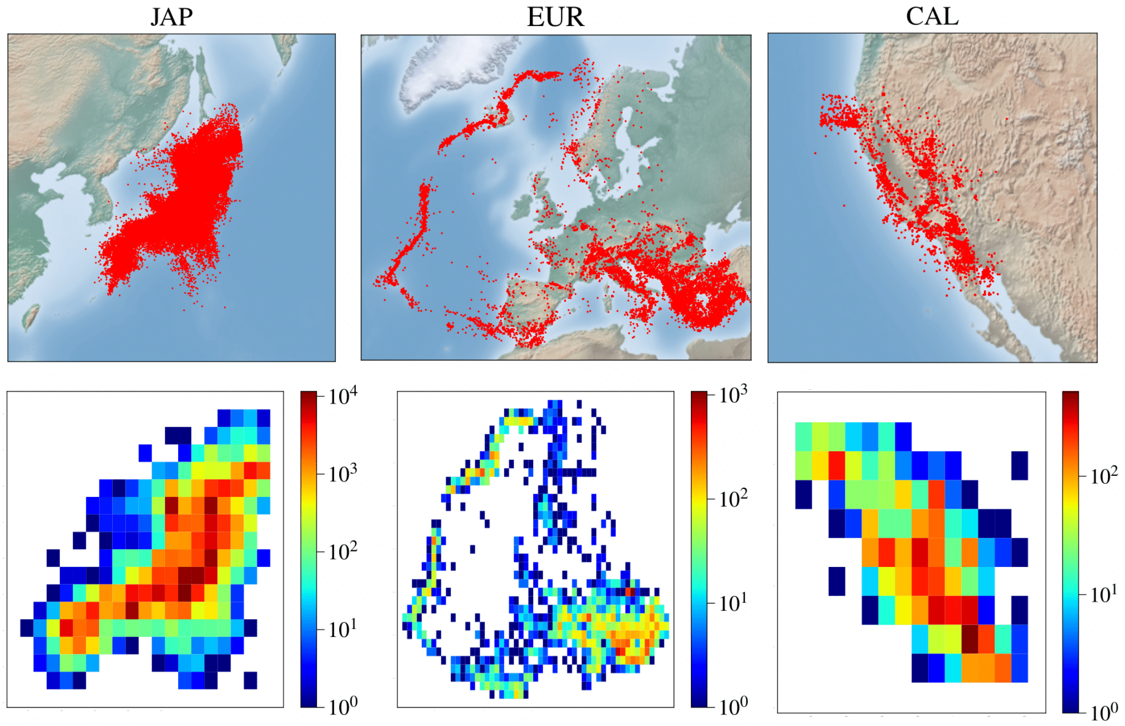

Figure S3: Construction of time series from seismological data. From left to right we report: the Japan catalog, the European catalog and the Californian catalog. Top row: spatial distribution of earthquakes in the three catalogs considered. Bottom row: histogram of the spatial distribution. Bins are squares of side 100 Km.

C Estimation of the exponents

To estimate the exponents $\hat{\tau}$ and $\hat{\alpha}$ for the empirical avalanche distributions, we use the fact that for a generic power-law probability distribution with exponent η, the maximum likelihood estimator can be written as

$$\hat{\eta} = 1 + Z \left(\sum_{i=1}^{Z} \ln \frac{x_i}{x_{min}} \right)^{-1} , \tag{S1}$$

where x_i is a data point of the empirical sample, and x_{min} is the smallest value of the sample that is expected to truly respect the power-law statistics [19]. Z is the number of data points

Data set	Acronym	Temporal window	Time series	Events	URL
Rat	RHDC	3,578,396.8 [ms]	88	876,629	[15]
Mouse	MSOS	1 [hour]	166	938,018	[16]
Macaque	MPC	174,890 [ms]	46	273,244	[17]
Japan	JAP	Jul. 1, 1985 - Dec. 31, 1998	192	199,446	[11]
California	CAL	Apr. 30, 1900 - Dec. 27, 2000	81	5,340	[12]
Europe	EUR	Jan. 8, 1900 - Dec. 31, 2006	638	19,126	[18]

Table S4: Summary table of data sets describing neuronal and seismological activity. From left to right we report: the name of the data set, the acronym we use to refer to the data set, the temporal window of data collection, the total number of time series, the total number of events (times) and a link to the original data.

$x_i \geq x_{min}$. If the variable under consideration is discrete, the factor x_{min} in the denominator of the logarithm in Eq. (S1) must be replaced by $x_{min} - 0.5$. The error on the maximum likelihood estimator is $\Delta\hat{\eta} = (\hat{\eta} - 1)/\sqrt{Z}$. We use $S_{min} = 2$ to fit the size distribution and $T_{min} = 2\Delta^*$ to fit the duration distribution. This protocol allows us to measure $\hat{\tau}$ and $\hat{\alpha}$ of the distributions $P(S)$ and $P(T)$, respectively, and to further measure the scaling exponent $\hat{\gamma}$ as $(\hat{\alpha} - 1)/(\hat{\tau} - 1)$. Assuming that the two estimators are uncorrelated, the uncertainty on the ratio $f(\hat{\tau}, \hat{\alpha}) = (\hat{\alpha} - 1)/(\hat{\tau} - 1)$ can be simply evaluated as $\sqrt{\left(\frac{\partial f}{\partial \hat{\tau}} \Delta\hat{\tau}\right)^2 + \left(\frac{\partial f}{\partial \hat{\alpha}} \Delta\hat{\alpha}\right)^2}$.

To independently estimate the exponent $\hat{\gamma}$, we take the logarithm of both sides in the equation $\langle S \rangle(T) \propto T^{\gamma}$ and perform linear regression. The exponent γ and its uncertainty are then given by $\hat{\gamma} = \frac{\sum_i (X_i - \langle X \rangle)(Y_i - \langle Y \rangle)}{\sum_i (X_i - \langle X \rangle)^2}$ and $\Delta\hat{\gamma} = \sqrt{\frac{\frac{1}{Z-2}\sum_i \epsilon_i^2}{\sum_i (X_i - \langle X \rangle)^2}}$ respectively, where $X = \log T$ and $Y = \log\langle S \rangle$, and ϵ are the residuals.

Table S6 reports the results of this analysis, as they are shown in Fig. 2 of the main manuscript.

Data set	RHDC	MSOS	MPC	JAP	CAL	EUR
Δ^* (s)	4.841	10.116	$1.188 \cdot 10^{-3}$	994,194	1,566,860	1,678,770

Table S5: Summary table of the values of Δ^* obtained by maximizing the susceptibility on data sets not representative of social media. We report the name of the data set (upper row) and the associated value of Δ^* (bottom row), expressed in seconds.

Data set	$\hat{\tau}$	$\hat{\alpha}$	$\hat{\gamma}$
TWT	2.2601 ± 0.0002	2.3716 ± 0.0007	1.45 ± 0.02
TLG	2.2187 ± 0.0004	2.435 ± 0.003	1.31 ± 0.03
PARL	2.010 ± 0.001	2.339 ± 0.003	1.37 ± 0.02
WEI	2.316 ± 0.001	2.743 ± 0.005	1.26 ± 0.05
STCK	2.2349 ± 0.0007	2.535 ± 0.002	1.31 ± 0.02
DEL	2.304 ± 0.002	2.381 ± 0.005	1.25 ± 0.01

Table S6: Summary table of the exponents estimators. From left to right, we report the name of the dataset, the maximum likelihood estimators $\hat{\tau}$ and $\hat{\alpha}$ and the scaling exponent $\hat{\gamma}$.

D Scaling in neuronal systems and earthquakes

We perform on the supplementary data sets the same analysis performed in the main text for data sets concerning social media. Results are shown in Fig. S4. The three data sets describing neuronal brain activity in different animals all display the branching process (BP) statistics for both the size and the duration distributions. The finding is consistent with previous studies [20, 21, 22]. The scaling relation between $\langle S \rangle$ and T does not show the scaling $\gamma = 2$ as expected from BP theory. However, a slightly superlinear relation between these quantities has been reported for many different neuronal systems [21, 23].

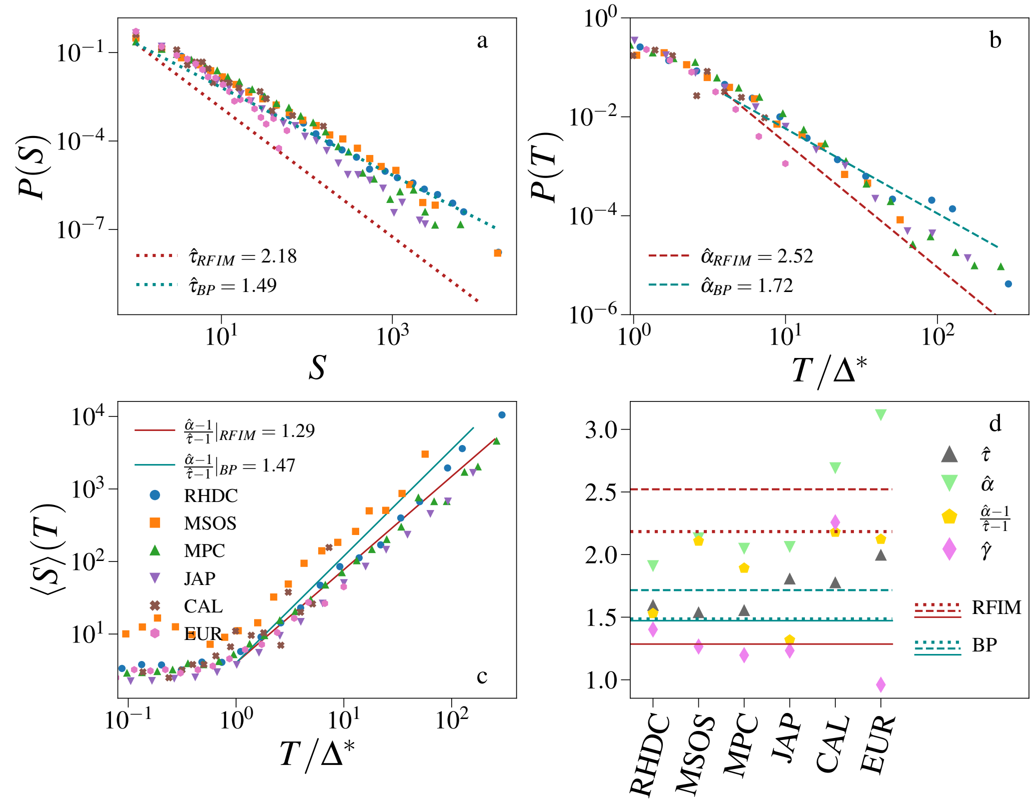

Figure S4: Avalanche statistics in systems other than social media. a) Distribution of avalanche size. Different colors/symbols refer to different systems: rat's hippocampal dissociated cultures (RHDC), mouse somatosensory organotypic slices (MSOS), macaque premotor cortex (MPC), earthquakes in Japan (JAP), California (CAL) and Europe (EUR). Dotted lines represent the maximum likelihood estimators of the exponent τ obtained by fitting the Random Field Ising Model (RFIM), in red, and the Branching Process (BP), in teal. The RFIM was fitted using $N = 10^9$, $R = 0.8$ and considering the same number of avalanches as the Twitter sample. The BP was fitted using $n = 1.0$ and sampling 10^6 avalanches. Distributions are displayed via logarithmic binning of the data. b) Distribution of avalanche duration for the same data as in panel a. Duration is rescaled by the factor $1/\Delta^*$ and probabilities are rescaled by the factor Δ^*. Dashed lines represent the maximum likelihood estimators of the exponent α obtained by fitting the RFIM (red) and the BP (teal) c) Average size of avalanches with given duration. Data are the same as in a and b. The abscissa of each curve is rescaled by $1/\Delta^*$. Solid lines represent the hyperscaling exponent $(\alpha - 1)/(\tau - 1)$ obtained using the maximum likelihood estimators of τ and α for the RFIM (red) and for the BP (teal). d) Maximum likelihood estimates of the exponents τ, α and γ, see SI G for details. We also display the ratio $(\hat{\alpha} - 1)/(\hat{\tau} - 1)$. Error bars are always smaller than the size of the symbol. Dotted lines correspond to the best fit of the exponent τ to the RFIM (red) and to the BP (teal), as shown in panel a. Analogously for dashed lines, representing the best fit of α as shown in b and for solid lines, representing the hyperscaling relation as shown in c.

E Temporal resolution and avalanche statistics

In Fig. S5 we show the complementary cumulative distribution of the data shown in Fig. 2 of the main text. In Fig. S6, we display the avalanche statistics of different systems obtained for different values of the temporal resolution Δ. For $\Delta \neq \Delta^*$, the power-law scaling is affected by apparent exponential cutoffs. The finding is in perfect agreement with theoretical arguments [24]. As the avalanche statistics obtained with the present approach represents the correlations existing in the system [14], the observation of distorted distributions means that the correlations existing in the data have not been properly identified. The same issue arises when each time series is assumed to be a unique avalanche, see Fig. S7.

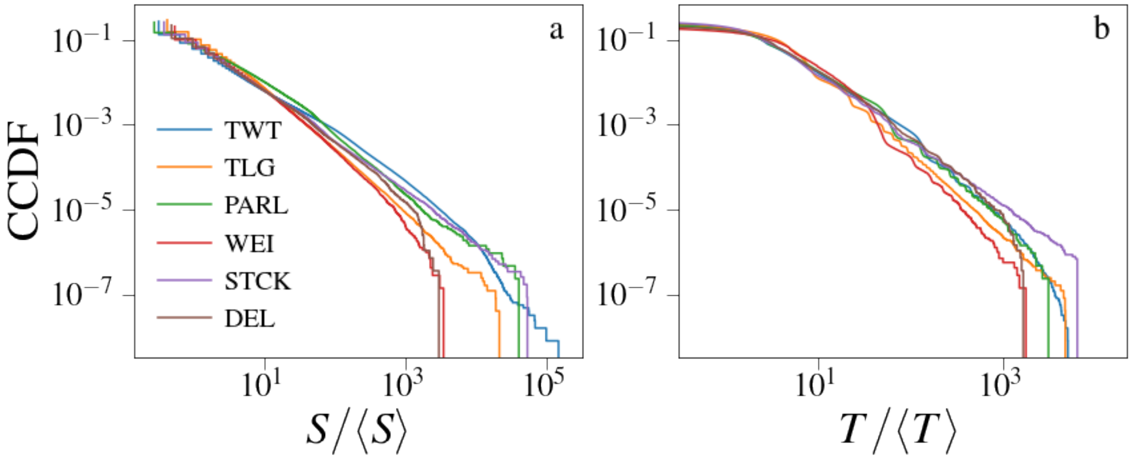

Figure S5: Complementary Cumulative Distribution Function of avalanche size and duration. We show the Cumulative Cumulative Distribution Function of avalanche size (panel a) and duration (panel b) for the six social media considered in our manuscript. The data in panel a are shown as a function of $S/\langle S\rangle$ and data in panel b are shown as a function of $T/\langle T\rangle$.

F The scaling function in the distribution of avalanche duration

The scaling function $\mathcal{D}_T$ appearing in the equation $P(T) = T^{-\alpha}\mathcal{D}_T(T^{1/z\nu}n')$ quickly goes to a constant value that is independent of T in the limit of large values of its argument, so that

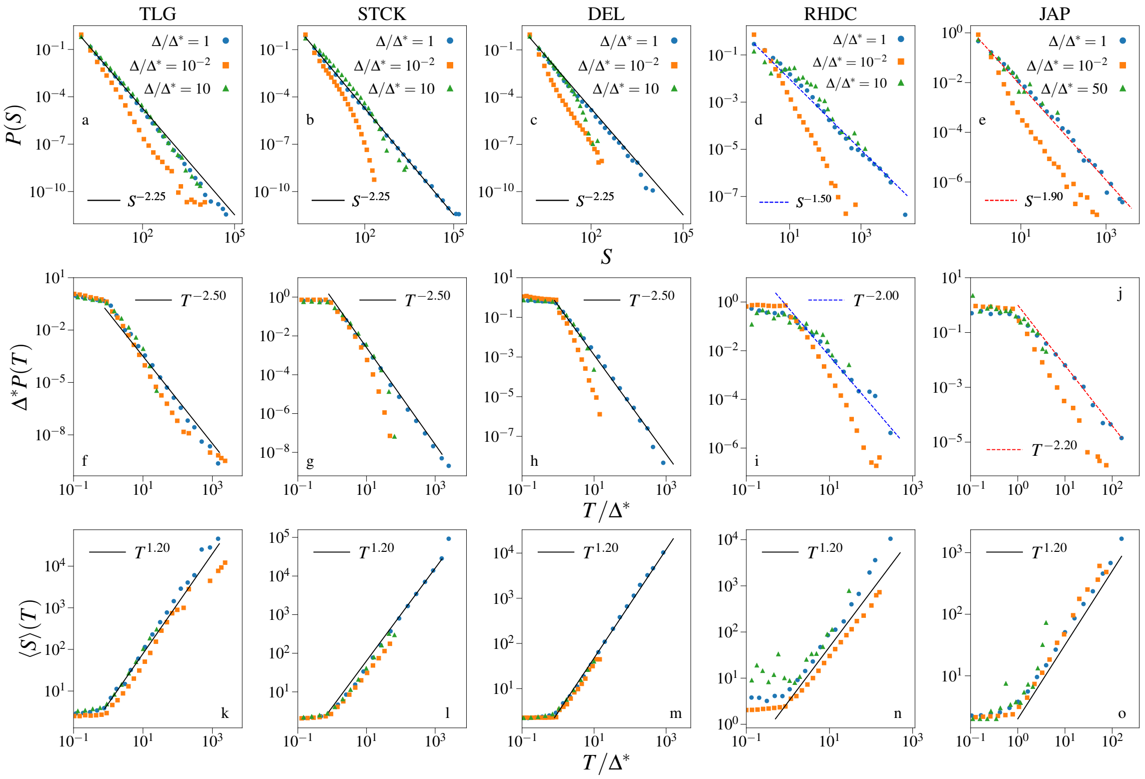

Figure S6: Statistics of avalanches obtained using different values of the temporal resolution. From left to right we show Telegram (TLG), Stackoverflow (STCK), Delicious (DEL), rat's hippocampal dissociated cultures (RHDC) and earthquakes in Japan (JAP). From top to bottom we show the avalanche size distribution, the avalanche duration distribution and the average size of avalanches with given duration. The abscissa in the second and third rows is rescaled by the factor $1/\Delta$ and the ordinate in the second row is rescaled by the factor Δ. Solid black lines represent the scaling reported for social media in the main text, i.e., $\tau = 2.25, \alpha = 2.5, \gamma = 1.2$, the dashed blue line represent the BP scaling, i.e., $\tau = 1.5, \alpha = 2$, and the dashed red lines represent the scaling $\tau = 1.9$ and $\alpha = 2.2$.

$P(T)$ shows the pure power-law decay $T^{-\alpha}$ in such a regime. The scaling function, however, introduces a correction to the pure power-law scaling at small values of its argument. As stated in the main text, the correction is rather strong for the RFIM in large dimension. The same phenomenology is experienced by the distribution of avalanche sizes $P(S)$, but the correction is much smaller in this case. Fig. S8 shows that the correction on $P(S)$ is nearly 3, while

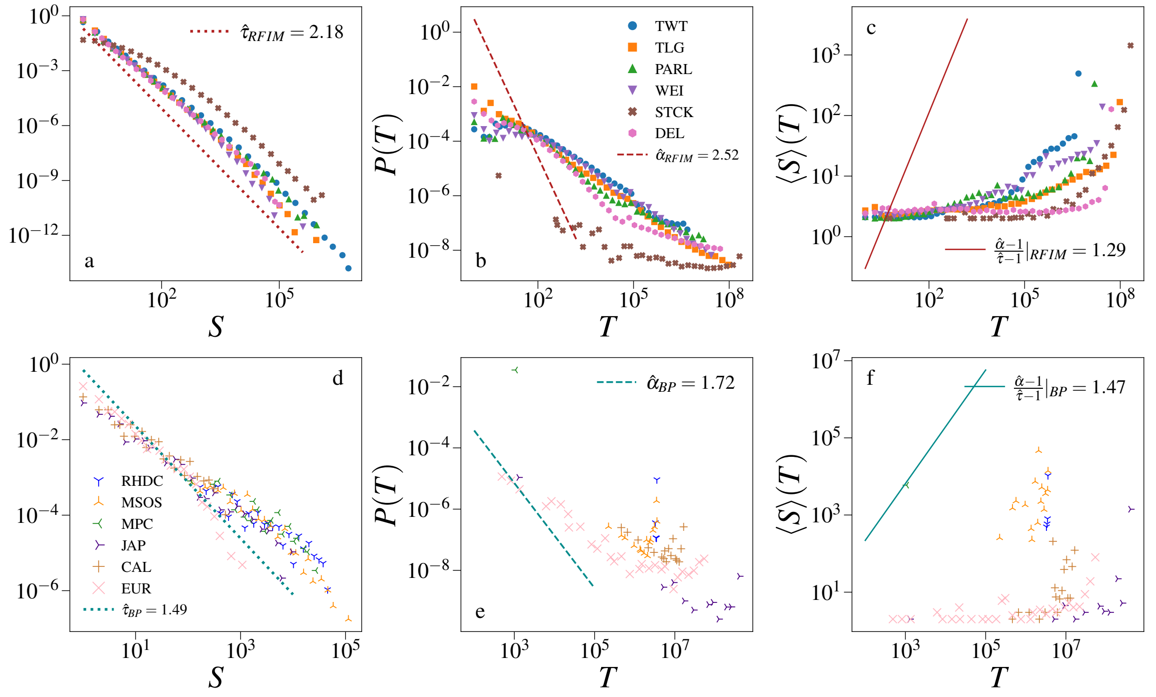

Figure S7: Avalanche statistics assuming each time series is an avalanche. a) Avalanche size distribution in social media. The dotted red line corresponds to best fit of the exponent $\hat{\tau}$ to the RFIM. b) Avalanche duration distribution for the same data as a. The dashed red line corresponds to best fit of the exponent $\hat{\alpha}$ to the RFIM. c) Average size of avalanches with given duration for the same data as A. The solid red line corresponds to best fit of the exponent $(\hat{\alpha} - 1)/(\hat{\tau} - 1)$ to the RFIM. d) Avalanche size distribution in systems other than social media. The dotted teal line corresponds to best fit of the exponent $\hat{\tau}$ to the BP. e) Avalanche duration distribution for the same data as D. The dashed teal line corresponds to best fit of the exponent $\hat{\alpha}$ to the BP. f) Average size of avalanches with given duration for the same data as D. The solid teal line corresponds to best fit of the exponent $(\hat{\alpha} - 1)/(\hat{\tau} - 1)$ to the BP.

the correction on $P(T)$ is larger than 50, the correction being computed as the ratio between the maximum of the scaling function and its value in the limit of small argument. Note that the correction on $P(S)$ in dimension 3 is about 10, and this is already sufficient to lead to an inaccurate estimation of the asymptotic exponent value [25]. Fig. S8 C and F further shows how this correction affects the measure of γ.

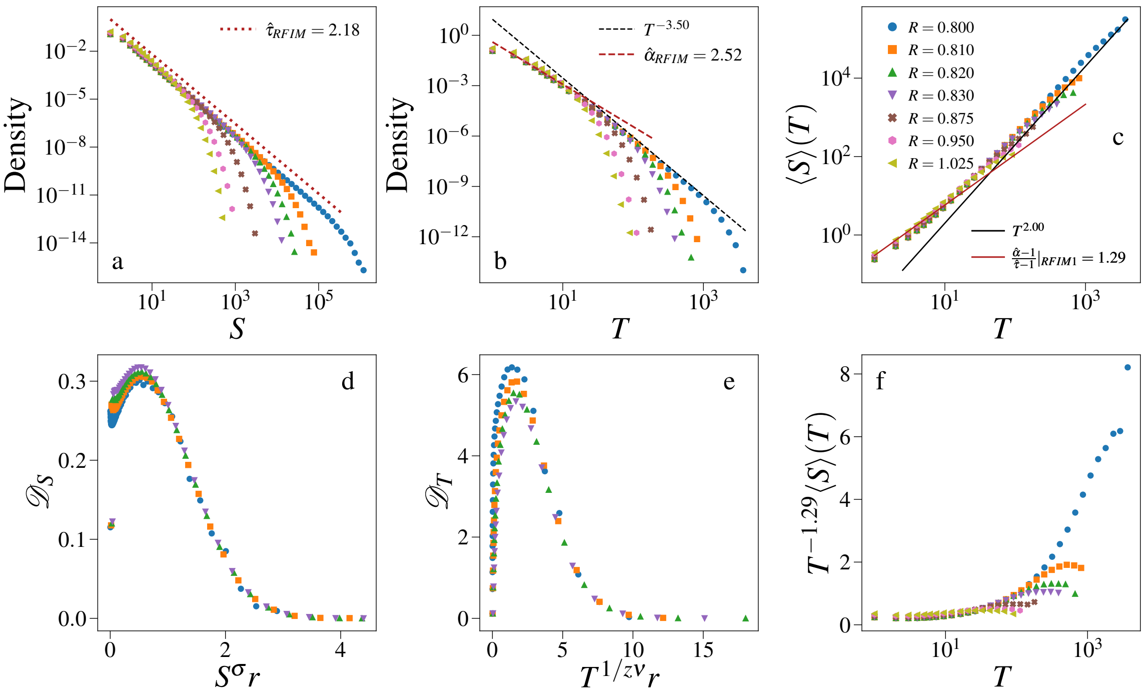

Figure S8: Avalanche statistics in the RFIM on a complete graph of size $N = 10^9$. a) Avalanche size distribution. The dotted red line corresponds to best fit of the exponent $\hat{\tau}$ to the RFIM. b) Avalanche duration distribution. The dashed black line scales as $T^{-\alpha}$ with $\alpha = 3.5$, while the dashed red line corresponds to the best fit of the exponent $\hat{\alpha}$ to the RFIM. c) Average size of avalanches with given duration. The solid black line scales as T^{γ} with $\gamma = 2$, while the solid red line corresponds to best fit of the exponent $(\hat{\alpha}-1)/(\hat{\tau}-1)$ to the RFIM. d) Scaling function of the size distribution. e) Scaling function of the duration distribution. f) Average size of avalanches with given duration, rescaled by $T^{-\hat{\tau}_{RFIM}}$.

G Calculation of the likelihood

The model distribution Q is estimated from numerical simulations. As such, finite-size distortions may be present in the tail of the empirical distribution, potentially leading to mistakes in the maximum likelihood fit. We therefore apply a rectangular kernel to regularize the empirical distribution. The width of the rectangular kernel grows exponentially with respect to its argument value at rate h. In Fig. S9, we show the distributions Q before and after smoothing for several configurations of both RFIM And BP. The analysis is performed by setting $h = 0.1$.

This is the value of the smoothing parameter used to obtain the results in the main text. We verified that results are robust against small variations of h, e.g., $h = 0.2$ or $h = 0.05$. Note that a small variation of h is a significant variation for the width of the rectangular kernel.

Once the smoothing is performed, the smoothed distributions represent the theoretical model. As such, we use the smoothed distributions for the calculation of the likelihood, for the calculation of the Kolmogorov-Smirnov (KS) distance, and for the generation of the synthetic samples required to estimate the p-value.

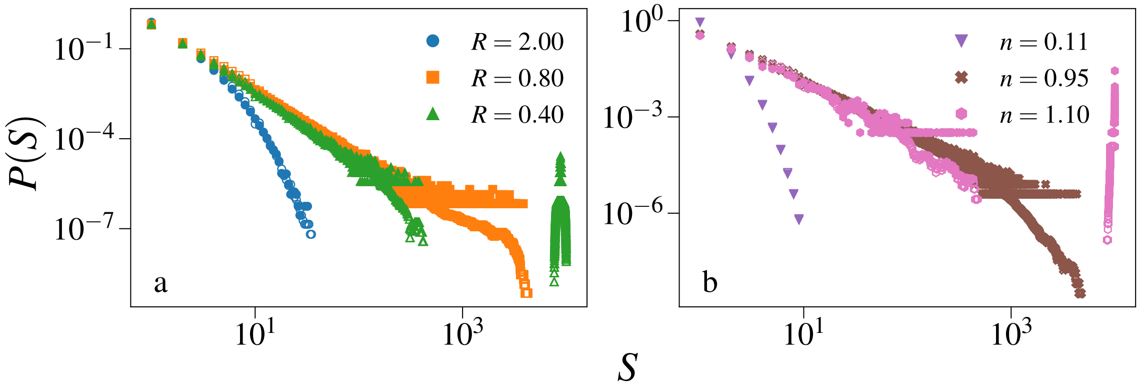

Figure S9: Avalanche size distributions before and after smoothing. In all panels, full symbols represent the original distribution and empty symbols represent the distribution after smoothing. We use here $h = 0.1$ and set the system size to be $N = 10^4$. a) Three configurations of the RFIM. b) Three configurations of the BP. Each panel shows a subcritical, a nearly critical and a supercritical configuration.

H Efficient computation of the KS distance

Let us indicate with C_P and C_Q the Cumulative Distribution Functions (CDFs) of the distributions P and Q, respectively. The Kolmogorov-Smirnov (KS) distance between P and Q is defined as

$$\mathrm{KS}(C_P, C_Q) = \max_y |C_P(y) - C_Q(y)| \,. \tag{S2}$$

To speed up the computation of Eq. (S2), we rewrite it as

$$KS(C_P, C_Q) = \max_{i=0,...,Z}\{|C_P(S_i) - C_Q(S_i)|, |C_P(S_{i+1} - 1) - C_Q(S_{i+1} - 1)|\}\,. \qquad \text{(S3)}$$

In the above equation, $1 \leq S_1 < S_2 < ...S_i < S_{i+1} < ... < S_Z \leq N$ are the sizes of the avalanches used to construct the empirical distribution P. By definition of CDF, we have that $C_P(S_i) < C_P(S_{i+1})$ for all $i = 0, ..., Z$, where we relied on the conventions $S_0 = -\infty$ and $S_{Z+1} > S_Z$, thus $C_P(S_0) = 0$ and $C_P(S_{Z+1}) = 1$.

Estimating the KS distance via Eq. (S3) requires to compute the difference between C_P and C_Q for a number of values of their arguments that is (much) smaller than the one required by the straight implementation of Eq. (S2), since $\{S_i\}$ is a subset of $\{y\}$ containing only the observed values of S.

To prove that Eq. (S3) holds we need to show that, for each $i = 0, \ldots, Z$, we have that

$$\max_{y\in[S_i,S_{i+1}-1]}|C_P(y) - C_Q(y)| = \max\{|C_P(S_i) - C_Q(S_i)|, |C_P(S_{i+1} - 1) - C_Q(S_{i+1} - 1)|\}\,. \qquad \text{(S4)}$$

The validity of the above equation follows from the facts that both C_P and C_Q are non-decreasing functions, and that C_P is constant in the interval $[S_i, S_{i+1} - 1]$. As a matter of fact, Eq. (S4) is representative for the only three possible cases that can happen:

1. $C_P(S_i) \leq C_Q(y)$ for each $y \in [S_i, S_{i+1} - 1]$. Then, for each y in this interval,

   $$|C_P(y) - C_Q(y)| = C_Q(y) - C_P(S_{i+1} - 1) \leq C_Q(S_{i+1} - 1) - C_P(S_{i+1} - 1)\,, \qquad \text{(S5)}$$

   so that

   $$\max_{y\in[S_i,S_{i+1}-1]}|C_P(y) - C_Q(y)| = |C_P(S_{i+1} - 1) - C_Q(S_{i+1} - 1)|\,. \qquad \text{(S6)}$$

2. $C_P(S_i) \geq C_Q(y)$ for each $y \in [S_i, S_{i+1} - 1]$. Then, for each y in this interval,

   $$|C_P(y) - C_Q(y)| = C_P(S_i) - C_Q(y) \leq C_P(S_i) - C_Q(S_i)\,, \qquad \text{(S7)}$$

so that

$$\max_{y\in[S_i,S_{i+1}-1]}|C_P(y)-C_Q(y)| = |C_P(S_i)-C_Q(S_i)|\,. \tag{S8}$$

3. $\exists\, y^* \in [S_i, S_{i+1}-1]$ such that $C_P(S_i) \geq C_Q(y)$ for $y \in [S_i, y^*]$ and $C_P(S_i) \leq C_Q(y)$ for $y \in [y^*, S_{i+1}-1]$. In this case, the interval $[S_i, y^*]$ can be treated as the former case 2 while the interval $[y^*, S_{i+1}-1]$ can be treated as the former case 1, so that in the present case 3 we have

$$\max_{y\in[S_i,S_{i+1}-1]}|C_P(y)-C_Q(y)| = \max\{|C_P(S_i)-C_Q(S_i)|, |C_P(S_{i+1}-1)-C_Q(S_{i+1}-1)|\}\,. \tag{S9}$$

I Validation on synthetic samples

To validate our fitting procedure we apply it on synthetic distributions P generated by the RFIM or by the BP. The method must be able to distinguish effectively between these two models. To this aim, we fix the system size to be $N = 10^6$ and fit 10^4 realizations of each model. Results are shown in Fig. S10. The fitting procedure is able to identify the ground truth, either RFIM or BP, regardless of the S_{min} value. In those cases in which model selection requires the log-likelihood ratio test, it still generally holds that the true model is selected with higher chances. In the case of synthetic data we can also compare the inferred parameter with the ground truth and Fig. S10 C and F show that the probability that these two quantities differ decays quickly as the difference departs from zero.

J Robustness of the fits

In the main text we show results of the fitting protocol using $S_{min} = 10$. Further, we estimated statistical significance by setting the threshold value to 0.1. Our conclusions, however, are unaffected by different choices of these parameters. In Fig. S11 and Fig. S12, we vary the threshold over the p-value and S_{min}, respectively.

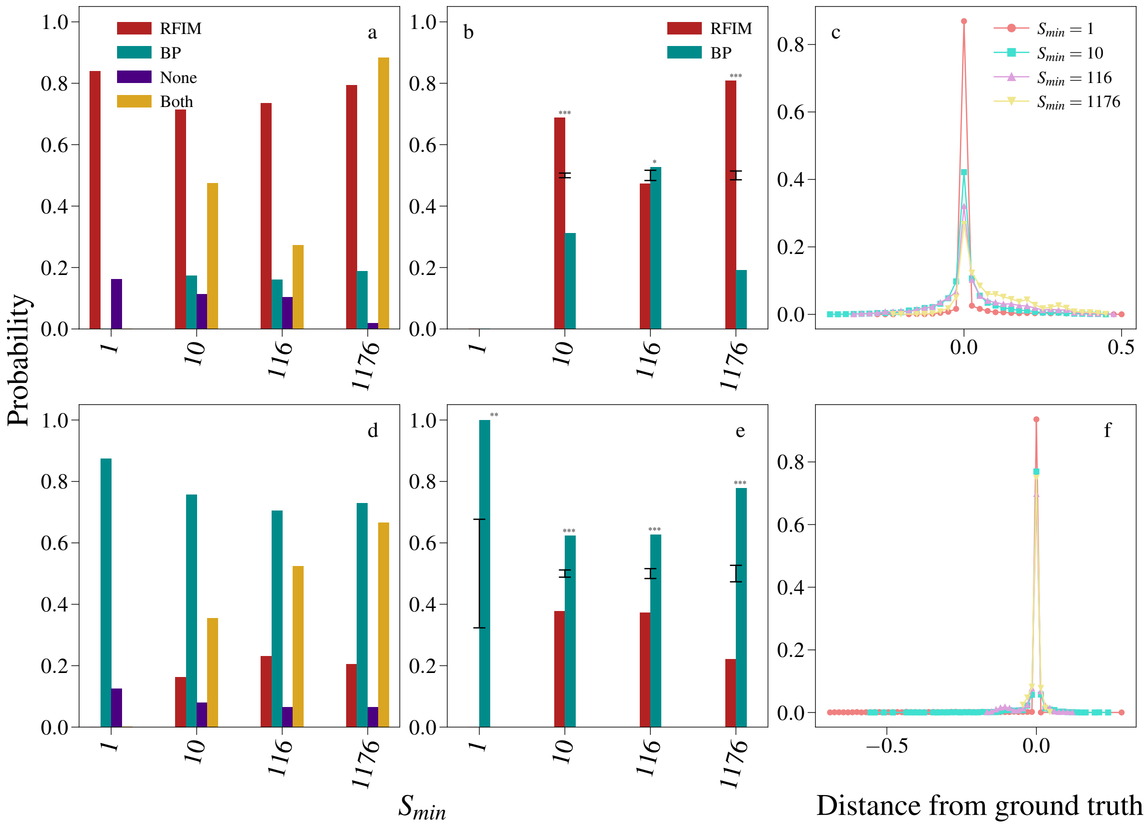

Figure S10: Fitting procedure applied to synthetic data sets. We show the results obtained when the RFIM is the ground truth (upper row) and when the BP is the ground truth (bottom row), considering four values of S_{min}. Left column: we report the overall probability that the RFIM (red) or the BP (blue) is the selected model, the probability that both models are discarded (purple) and the probability that both the models are not individually rejected so that the model selection is performed by means of the log-likelihood ratio test (yellow). Central column: we report the probability that the RFIM (red) or the BP (blue) is the model selected by means of the log-likelihood ratio test. Error bars represent σ/N, where N is the sample size and $\sigma = \sqrt{0.25N}$ is the standard deviation of a binomial distribution with probability of success equal to $1/2$. Asterisks are used to denote significant deviations from the unbiased binomial model, i.e., three asterisks indicate for $p < 0.001$. Right column: we report the probability distribution of the distance between the true value of the parameter used to generate the distribution P and the parameter inferred by fitting against the true model.

K Hashtags in the simple and complex contagion classes

We report here a table containing the 30 worlds shown in the word cloud of Fig. 4 of the main text, ranked according to their popularity.

Rank	RFIM class	BP class
1	ThursdayThoughts	GOT7_CallMyName
2	AgustD	exo
3	PowerTV	AMAs
4	clip	TheGroup
5	sao_anime	POPTimeAwards
6	Caturday	Bolivia
7	anipoke	seventeen
8	RunAway	TheMusicVideo
9	ChampionsLeague	TheConcertTour
10	SundayMotivation	MakeItRight
11	HereWeGo	RWC2019
12	ONCE	AyKer
13	TEAMFOLLOWBACK	MMA2019
14	SNL	peing
15	ChileDespierta	SarileruNeekevvaru
16	MRpoints	Viswasam
17	MV	KristPerawat
18	Legacies	AFazenda11
19	weekend	GoHabsGo
20	SmashBrosUltimate	WINNER
21	CowboysNation	FightForWonho
22	RavensFlock	Chile
23	ThisIsUs	SingtoPrachaya
24	Changbin	SpeakYourselfTourFinal
25	tiktok	imgxnct
26	Singapore	LALISA
27	911onFOX	GOT7_KEEPSPINNING
28	HKHumanRightsandDemocracyAct	BTS_POPUP
29	AyodhyaCase	Mark
30	YOONGI	ModoActivo

Table S7: Summary table of the 30 most popular hashtags in the two dynamic classes. From left to right we report the rank of hashtag, the hashtag in the complex contagion class with that rank and the hashtag in the complex simple class with that rank. Rank is proportional to the number of events in the time series. The hashtags reported here are the same shown in the word cloud of Fig. 4 of the main text.

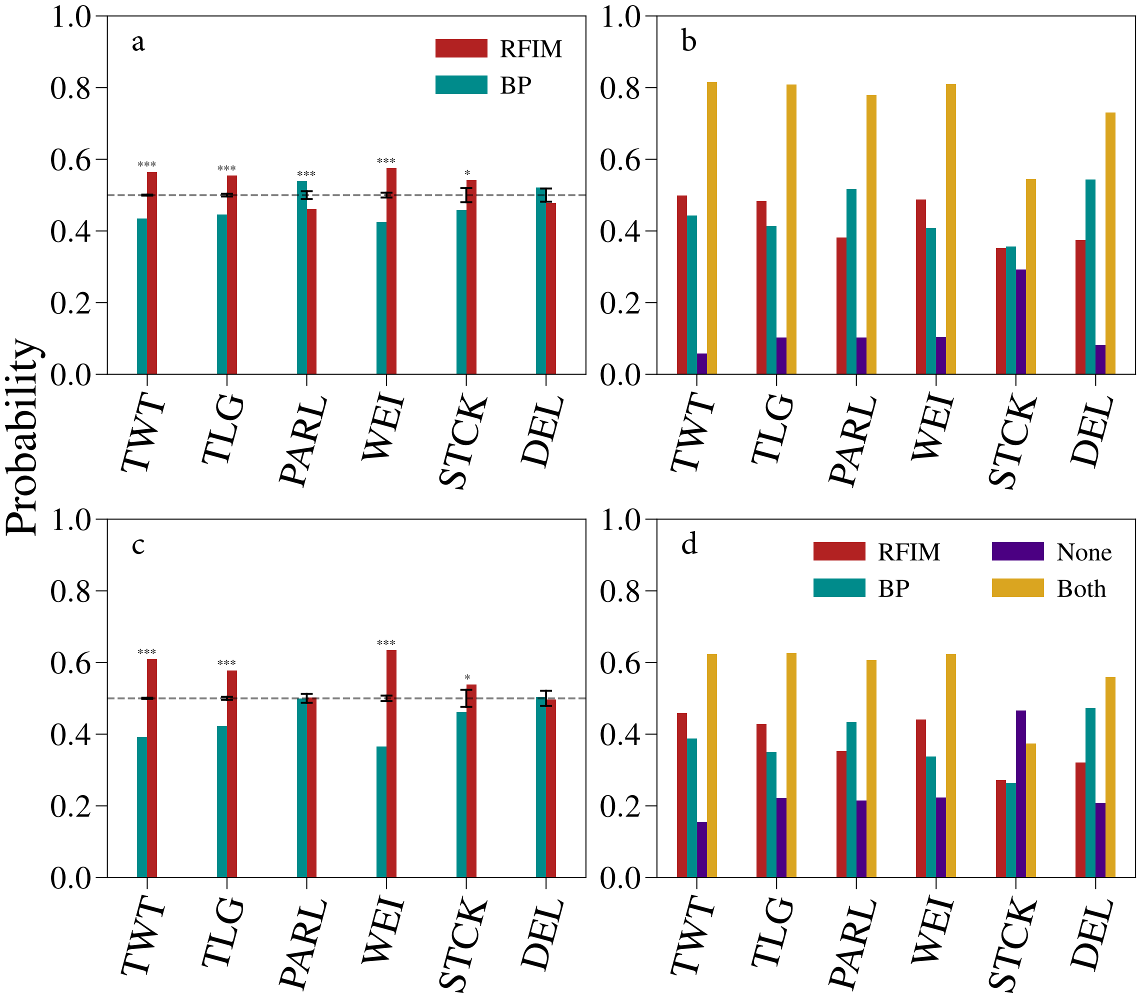

Figure S11: Robustness against the level of statistical significance. We set statistical significance equal to 0.05 (upper row) and 0.2 (lower row). Acronyms of the data sets are the same used in the main text. a) Probability that the log-likelihood ratio test favors RFIM over BP (blue), or vice versa BP over RFIM (red), using a threshold 0.05 over the p values. Only time series that are sufficiently well fitted by both models are considered in the analysis, see panel b. Error bars represent σ/N, where N is the sample size and $\sigma = \sqrt{0.25N}$ is the standard deviation of a binomial distribution with probability of success equal to $1/2$. Asterisks are used to denote significant deviations from the unbiased binomial model, i.e., two asterisks indicate for $p < 0.01$ and one asterisk stands for $p < 0.1$. b) We report the fraction of time series that are classified in the RFIM class (red), the fraction of time series that are classified as BP (blue), the fraction of time series that is classified as neither BP nor RFIM (purple) and the fraction of time series that pass both statistical tests (yellow). In this case, the log-likelihood ratio test is required for model selection, see panel a. Here we set to 0.05 the threshold over the p values. c) Same as in panel a, but the threshold over the p values is set to 0.2. d) Same as in panel b, but the threshold over the p values is set to 0.2.

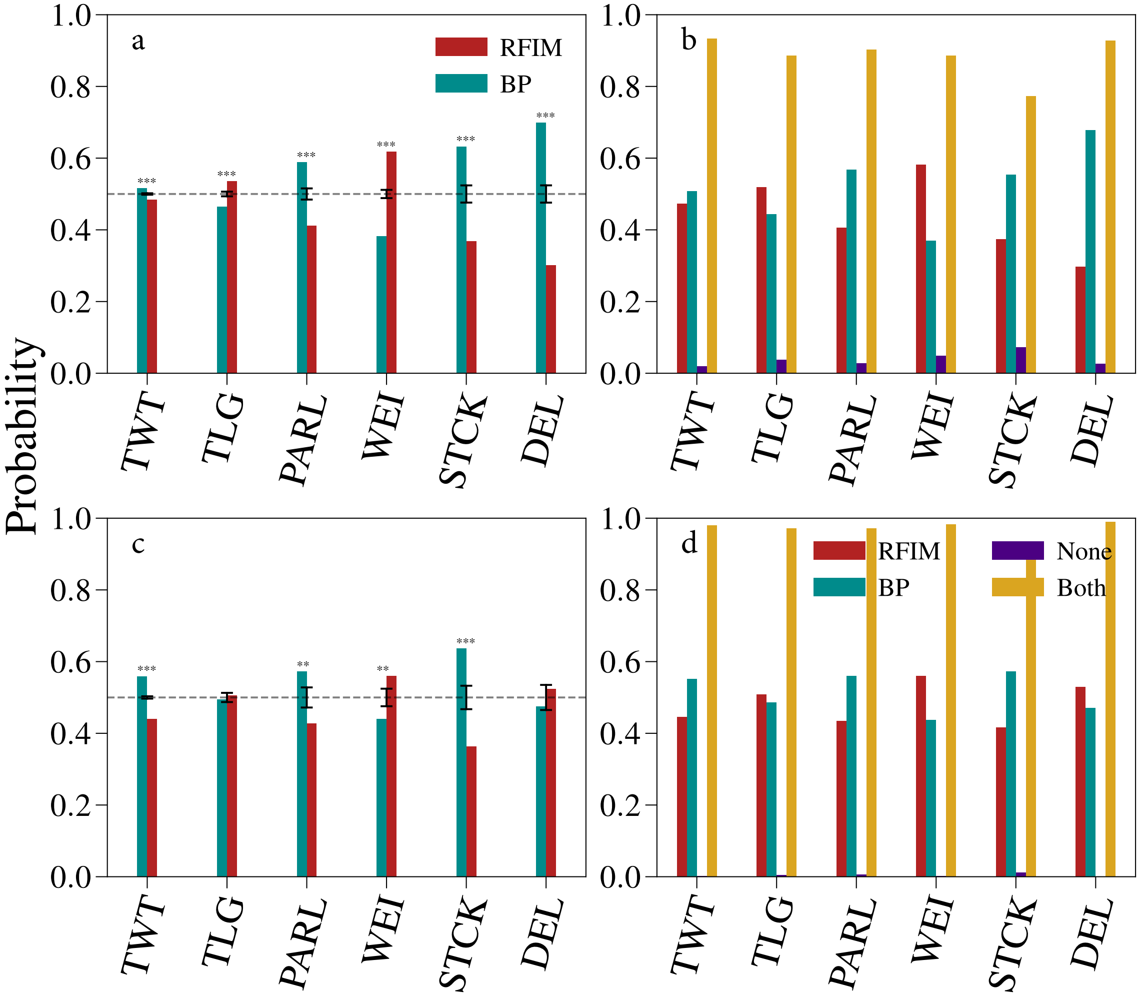

Figure S12: Robustness against S_{min} values. Here we use $S_{min} = 27$ (upper row) and $S_{min} = 95$ (lower row). Acronyms of the data sets are the same used in the main text. a) Probability that the log-likelihood ratio test favors RFIM over BP (blue), or vice versa BP over RFIM (red), using $S_{min} = 27$. Only time series that are sufficiently well fitted by both models are considered in the analysis, see panel b. Error bars represent σ/N, where N is the sample size and $\sigma = \sqrt{0.25N}$ is the standard deviation of a binomial distribution with probability of success equal to $1/2$. Asterisks are used to denote significant deviations from the unbiased binomial model, i.e., two asterisks indicate for $p < 0.01$ and one asterisk stands for $p < 0.1$. b) We report the fraction of time series that are classified in the RFIM class (red), the fraction of time series that are classified as BP (blue), the fraction of time series that is classified as neither BP nor RFIM (purple) and the fraction of time series that pass both statistical tests (yellow). In this case, the log-likelihood ratio test is required for model selection, see panel a. Here we use $S_{min} = 27$. c) Same as in panel a, but $S_{min} = 95$. d) Same as in panel b, but $S_{min} = 95$.

Supplementary References

[1] Notarmuzi, D., Castellano, C., Flammini, A., Mazzilli, D. & Radicchi, F. https://github.com/DaniMuzi/SocialMedia (2021).

[2] Baumgartner, J., Zannettou, S., Squire, M. & Blackburn, J. https://zenodo.org/record/3607497#.YRu-4tMza-s (2020).

[3] Aliapoulios, M. *et al.* https://zenodo.org/record/4442460#.YRu_WtMza-s (2021).

[4] Fu, K.-w. Weiboscope open data. (dataset). https://hub.hku.hk/cris/dataset/dataset107483 (2017).

[5] Link to stackoverflow data. https://archive.org/download/stackexchange/stackoverflow.com-Posts.7z.

[6] Basile, V. http://valeriobasile.github.io/delicious/ (2015).

[7] Timme, N. M. *et al.* Criticality maximizes complexity in neural tissue. *Front. Physiol.* **7**, 425 (2016).

[8] Ito, S. *et al.* Large-scale, high-resolution multielectrode-array recording depicts functional network differences of cortical and hippocampal cultures. *PLoS One* **9**, e105324 (2014).

[9] Litke, A. *et al.* What does the eye tell the brain?: Development of a system for the large-scale recording of retinal output activity. *IEEE Trans. Nucl. Sci.* **51**, 1434–1440 (2004).

[10] Lawlor, P. N., Perich, M. G., Miller, L. E. & Kording, K. P. Linear-nonlinear-time-warp-poisson models of neural activity. *J. Comput. Neurosci.* **45**, 173–191 (2018).

[11] http://wwweic.eri.u-tokyo.ac.jp/CATALOG/junec/monthly.html.

[12] California earthquakes (dataset). https://www.conservation.ca.gov/cgs/Documents/Melange/cgs2000_fnl.txt.

[13] Grünthal, G., Wahlström, R. & Stromeyer, D. The share european earthquake catalogue (sheec) for the time period 1900–2006 and its comparison to the european-mediterranean earthquake catalogue (emec). *J. Seismol.* **17**, 1339–1344 (2013).

[14] Karsai, M., Kaski, K., Barabási, A.-L. & Kertész, J. Universal features of correlated bursty behaviour. *Sci. Rep.* **2**, 1–7 (2012).

[15] Timme, N. M. *et al.* Dissociated cutlures of rat's hippocampal cells (dataset). https://crcns.org/data-sets/hc/hc-8 (2016).

[16] Ito, S. *et al.* Spontaneous spiking activity of hundreds of neurons in mouse somatosensory cortex slice cultures recorded using a dense 512 electrode array. http://dx.doi.org/10.6080/K07D2S2F (2016).

[17] Perich, M. G., Lawlor, P. N., Kording, K. P. & Miller, L. E. Extracellular neural recordings from macaque primary and dorsal premotor motor cortex during a sequential reaching task. http://dx.doi.org/10.6080/K0FT8J72 (2018).

[18] Grünthal, G., Wahlström, R. & Stromeyer, D. Data taken from sheec 1900-2006 (grünthal et al., 2013) . https://www.gfz-potsdam.de/sheec/ (2013).

[19] Clauset, A., Shalizi, C. R. & Newman, M. E. Power-law distributions in empirical data. *SIAM Rev.* **51**, 661–703 (2009).

[20] Beggs, J. M. & Plenz, D. Neuronal avalanches in neocortical circuits. *J. Neurosci.* **23**, 11167–11177 (2003).

[21] Friedman, N. *et al.* Universal critical dynamics in high resolution neuronal avalanche data. *Phys. Rev. Lett.* **108**, 208102 (2012).

[22] Haldeman, C. & Beggs, J. M. Critical branching captures activity in living neural networks and maximizes the number of metastable states. *Phys. Rev. Lett.* **94**, 058101 (2005).

[23] Fontenele, A. J. *et al.* Criticality between cortical states. *Phys. Rev. Lett.* **122**, 208101 (2019).

[24] Notarmuzi, D., Castellano, C., Flammini, A., Mazzilli, D. & Radicchi, F. Percolation theory of self-exciting temporal processes. *Phys. Rev. E* **103**, L020302 (2021).

[25] Sethna, J. P., Dahmen, K. A. & Myers, C. R. Crackling noise. *Nature* **410**, 242–250 (2001).
