# Supplementary material for: Universality, criticality and complexity of information propagation in social media
Source: Nat Commun. 2022 Mar 14;13:1308. doi: 10.1038/s41467-022-28964-8 (PMC8921196; doi:10.1038/s41467-022-28964-8)
Supplement: Supplementary file 3 — Reporting Summary [file 41467_2022_28964_MOESM3_ESM.pdf]

nature portfolio

Corresponding author(s): Filippo Radicchi

Last updated by author(s): Jan 25, 2022

Reporting Summary

Nature Portfolio wishes to improve the reproducibility of the work that we publish. This form provides structure for consistency and transparency in reporting. For further information on Nature Portfolio policies, see our Editorial Policies and the Editorial Policy Checklist.

Statistics

For all statistical analyses, confirm that the following items are present in the figure legend, table legend, main text, or Methods section.

n/a	Confirmed	
☐	☒	The exact sample size (*n*) for each experimental group/condition, given as a discrete number and unit of measurement
☐	☒	A statement on whether measurements were taken from distinct samples or whether the same sample was measured repeatedly
☐	☒	The statistical test(s) used AND whether they are one- or two-sided<br>*Only common tests should be described solely by name; describe more complex techniques in the Methods section.*
☒	☐	A description of all covariates tested
☐	☒	A description of any assumptions or corrections, such as tests of normality and adjustment for multiple comparisons
☒	☐	A full description of the statistical parameters including central tendency (e.g. means) or other basic estimates (e.g. regression coefficient) AND variation (e.g. standard deviation) or associated estimates of uncertainty (e.g. confidence intervals)
☐	☒	For null hypothesis testing, the test statistic (e.g. *F*, *t*, *r*) with confidence intervals, effect sizes, degrees of freedom and *P* value noted<br>*Give P values as exact values whenever suitable.*
☒	☐	For Bayesian analysis, information on the choice of priors and Markov chain Monte Carlo settings
☒	☐	For hierarchical and complex designs, identification of the appropriate level for tests and full reporting of outcomes
☒	☐	Estimates of effect sizes (e.g. Cohen's *d*, Pearson's *r*), indicating how they were calculated

Our web collection on statistics for biologists contains articles on many of the points above.

Software and code

Policy information about availability of computer code

Data collection	Twitter data were collected using the Twitter decahose stream, meaning a random 10% of the daily activity on Twitter were provided to us directly by Twitter. Datasets concerning other social media were not collected by the present authors.<br><br>Twitter decahose stream: https://developer.twitter.com/en/docs/twitter-api/enterprise/decahose-api/overview/decahose
Data analysis	No softwares were used.

For manuscripts utilizing custom algorithms or software that are central to the research but not yet described in published literature, software must be made available to editors and reviewers. We strongly encourage code deposition in a community repository (e.g. GitHub). See the Nature Portfolio guidelines for submitting code & software for further information.

Data

Policy information about availability of data

All manuscripts must include a data availability statement. This statement should provide the following information, where applicable:

- Accession codes, unique identifiers, or web links for publicly available datasets
- A description of any restrictions on data availability
- For clinical datasets or third party data, please ensure that the statement adheres to our policy

The Twitter data generated in this study have been deposited in the Zenodo and GitHub database.
Link to Zenodo repository: https://zenodo.org/record/5779063#.Yenws1jMLAJ
Link to GitHub repository: https://github.com/DaniMuzi/SocialMedia

Telegram, Parler, Weibo, Stackoverflow and Delicious data used in this study have been generated in other works. Urls to each of these datasets are provided in the Supplementary Information file (section A)

Field-specific reporting

Please select the one below that is the best fit for your research. If you are not sure, read the appropriate sections before making your selection.

- [ ] Life sciences
- [x] Behavioural & social sciences
- [ ] Ecological, evolutionary & environmental sciences

For a reference copy of the document with all sections, see nature.com/documents/nr-reporting-summary-flat.pdf

Behavioural & social sciences study design

All studies must disclose on these points even when the disclosure is negative.

Study description	We make a statistical analysis of time series. Data are quantitative.
Research sample	The research sample is a set of time series collected from Twitter, including all Twitter users without restrictions on age, gender, race, religion. The sample is the most complete dataset with twitter time series that we could create or use from third parties. The study also involves data from Telegram, Weibo, Parler, StackOverflow and Delicious originated in other studies. Datasets also come in the form of time series and the sources are other scientific publications (with the exception of StackOverflow, whose data come from a public repository).
Sampling strategy	The sampling of the Tweets consists of a random 10% of the daily activity on Twitter obtained via the Twitter decahose stream. Rather than predetermine the sample size, we predetermined the length of the temporal window of observation (two months). This choice allows for a large dataset and for long time series, while preserving the handling of the data.<br><br>Twitter decahose stream: https://developer.twitter.com/en/docs/twitter-api/enterprise/decahose-api/overview/decahose
Data collection	Twitter data were collected using the Twitter decahose stream, meaning a random 10% of the daily activity on Twitter were provided to us directly by Twitter, implying researchers were blind to the experimental condition and to the study hypothesis. Datasets concerning other social media were not collected by the present authors, which were thus blind to experimental conditions and to the study hypothesis.<br><br>Twitter decahose stream: https://developer.twitter.com/en/docs/twitter-api/enterprise/decahose-api/overview/decahose
Timing	The data were collected during the month of December 2019.
Data exclusions	No data were excluded from the analysis.
Non-participation	n/a
Randomization	Participants were not allocated in experimental groups.

Reporting for specific materials, systems and methods

We require information from authors about some types of materials, experimental systems and methods used in many studies. Here, indicate whether each material, system or method listed is relevant to your study. If you are not sure if a list item applies to your research, read the appropriate section before selecting a response.

Materials & experimental systems

n/a	Involved in the study	
x		Antibodies
x		Eukaryotic cell lines
x		Palaeontology and archaeology
x		Animals and other organisms
x		Human research participants
x		Clinical data
x		Dual use research of concern

Methods

n/a	Involved in the study	
x		ChIP-seq
x		Flow cytometry
x		MRI-based neuroimaging
